# EHA Endorsement of ESMO Clinical Practice Guidelines for Diagnosis, Treatment, and Follow-up for Myelodysplastic Syndromes

**DOI:** 10.1097/HS9.0000000000000695

**Published:** 2022-02-24

**Authors:** Arjan A. van de Loosdrecht, Inga Mandac Smoljanović

**Affiliations:** 1 Department of Hematology, Amsterdam UMC, VU University Medical Center, Cancer Center Amsterdam, The Netherlands; 2 Department of Hematology, Clinical Hospital Merkur, Zagreb, Croatia

European Hematology Association (EHA) and European Society for Medical Oncology (ESMO) recently agreed to collaborate on the production of European Guidelines for different hematological malignancies. As a first step, a number of completed guidelines have been reviewed by the corresponding EHA Scientific Working Groups in a standardized review process. The ESMO Clinical Practice Guidelines for diagnosis, treatment, and follow-up for myelodysplastic syndromes (MDS) published on November 19, 2020 (https://www.annalsofoncology.org/article/S0923-7534(20)43129-1/fulltext) in accordance with the ESMO standard operating procedures for clinical practice guidelines development were recently endorsed by the EHA.^1,2^

Fenaux et al provided an excellent and evidence-based overview of the state of the art guidelines of MDS. They covered and discussed the entire field of MDS by providing the latest data on incidence and epidemiology, diagnostic strategies, risk assessment, and treatment options in MDS. Evidence is provided by using the international used grading/Strength of Recommendation Taxonomy system for evidence. The document is supported by clear tables and figures supporting the text to allow for appropriate fast reading.

Myelodysplastic syndrome is a rare disease with an estimated incidence of 4 cases/100,000 persons/year with a median age at diagnosis of 70 years. It is well known that the incidence of MDS increases in patients older than 70 years, reaching an incidence of 40–50/100,000. It should be noted that the cause of MDS is only known in 15% of cases, with secondary MDS representing a group of patients with poor prognosis. The authors emphasize the complexity of diagnostic procedures. All available tests are mentioned and reviewed including emerging new platforms including next generation sequencing and multiparameter flow cytometry. Special emphasis is focused on definitions of potential pre-MDS conditions such as idiopathic cytopenia of undetermined significance, idiopathic dysplasia of undetermined significance, clonal hematopoiesis of indeterminate potential, and clonal cytopenia of undetermined significance. Final diagnosis and classification is based on the latest used models of the WHO 2016 and the Revised International Prognostic Scoring System (IPSS-R).^3,4^ It should be noted that the current WHO 2016 classification is under revision. Risk stratification according to the IPSS-R at diagnosis remains a major subject of interest in MDS due to its dynamic and sometimes unpredictable disease nature. Although there is a well-recognized subgroup of lower risk patients, the natural course of MDS is highly unpredictable. In addition, also within the intermediate and even higher IPSS-R risk-group, a watch and tightly wait approach might be justified stressing that additional factors may be taken into account. Finally, intermediate-risk MDS patients are the ones with more comorbidities, higher Eastern Cooperative Oncology Group scores and need for more individualized treatment approaches.

Regarding the treatment of higher risk MDS, the recommendations are based on evidence as well as on expert opinion if evidence by phase III clinical trials are lacking. All considerations which are common in routine clinical practice are discussed appropriately. As an example, the discussion on the value and type of induction therapy before treatment with an allogenic transplantation in cases with a blast number below or even >10% marrow blasts in MDS is not well established. Critical notes support the fact that to some extent evidence is still lacking. Regarding lower risk MDS, a treatment algorithm is provided and easy to follow, supported by either evidence or expert opinions. All recommendations are made after critical evaluation. Response criteria to treatment may depend on agents that modify disease course, improve cytopenia and have impact on quality of life. It is clear that there is an unmet need for new treatment options since current available agents are sparse. In particular, in higher risk MDS combinations with hypomethylating agents are disappointing without clear advantage. Despite 20 years of clinical trials in MDS, only a small number of drugs were successful in phase III clinical trials, among which azacitidine, decitabine, erythropoietin, lenalidomide, and recently luspatercept.^5^ This might be due to the lack of drugs specifically developed for MDS and lack of insights in MDS pathobiology. In conclusion, Fenaux et al provided an up-to-date guideline on MDS supported by well-balanced selection of arguments and evidence, which make this guideline suitable for daily clinical practice.

## AUTHOR CONTRIBUTIONS

AAvdL and IMS both equally wrote and approved the article.

## DISCLOSURES

The authors have no conflicts of interest to disclose.
